# Reviewing Mobile Dental Apps for Children with Cognitive and Physical Impairments and Ideating an App Tailored to Special Healthcare Needs

**DOI:** 10.3390/jcm14062105

**Published:** 2025-03-19

**Authors:** Massimo Pisano, Alessia Bramanti, Federica Di Spirito, Maria Pia Di Palo, Giuseppina De Benedetto, Alessandra Amato, Massimo Amato

**Affiliations:** 1 Department of Medicine, Surgery and Dentistry “Scuola Medica Salernitana”, University of Salerno, Via Salvador Allende, 84081 Baronissi, Italy; abramanti@unisa.it (A.B.); mariapia140497@gmail.com (M.P.D.P.); giusydb15@gmail.com (G.D.B.); mamato@unisa.it (M.A.); 2 Department of Neuroscience, Reproductive Science and Dentistry, University of Naples Federico II, 80131 Naples, Italy; aaleamato@gmail.com

**Keywords:** oral health, children, special needs, impairment, pedodontics, disability, mobile applications, teledentistry

## Abstract

**Background/Objectives**: Children with special healthcare needs (SHCNs) face various physical, developmental, sensory, behavioral, or cognitive challenges affecting oral health and highlighting the need for specialized and more accessible oral and dental care. Mobile health (m-health) applications have emerged as a promising solution to bridge oral health education gaps and improve dental care access. This narrative review aimed to examine the available dental m-health applications designed for children with cognitive and physical impairments, exploring the perspectives of children, parents/caregivers, and dentists regarding their use and potential contribution to oral health. Based on these insights, a concept for a tailored dental m-health app is proposed, considering the interactions between children, caregivers, and dentists to support oral care. **Methods**: A literature search was conducted using MEDLINE/PubMed, Scopus, and Web of Science to identify studies on the use of m-health apps in pediatric dentistry for SHCN children. **Results**: Six studies were included in this review. M-health applications helped manage anxiety, behavioral issues, and compliance in children and were generally well received by parents and caregivers, thus improving oral hygiene practices and regular dental visits, and having the potential to reduce oral health disparities in children with cognitive and physical impairments and their families. However, current apps designed for children are limited and may not fully accommodate the diverse sensory needs of these SHCN children. **Conclusions**: The development of a tailored dental m-health app that adapts to the individual characteristics of SHCN children could enhance oral health awareness and support better compliance among children, parents/caregivers, and dentists.

## 1. Introduction

Special healthcare needs (SHCNs) encompass a diverse range of physical, developmental, mental, sensory, behavioral, cognitive, or emotional impairments or limiting conditions necessitating specialized medical management, healthcare intervention, and multidisciplinary healthcare services or programs [1]. These conditions could be present from birth, developed over time, or acquired due to illness, injury, or environmental factors, and may result in limitations in performing daily self-maintenance activities or significant restrictions in major life activities [1].

Individuals classified as having SHCNs include those with congenital conditions such as trisomy 21 or heart malformations, developmental conditions like cerebral palsy or cognitive and intellectual disabilities, as well as systemic diseases or genetic syndromes [2]. SHCNs also include individuals with behavioral conditions such as anxiety, attention deficit and hyperactivity disorder (ADHD), or autism spectrum disorder (ASD) [2,3].

SHCN populations are highly heterogeneous, with differences in the severity of impairments, cognitive levels, and health maintenance capabilities. To address this variability, several classifications have been proposed. Frank and Winter identified eight categories of impairment, including sensory (e.g., blindness, deafness), physical (e.g., mobility impairments), cognitive (e.g., intellectual disabilities), behavioral (e.g., maladjustments), and speech-related [4,5]. Agerholm distinguished between intrinsic and extrinsic disabilities, while Novak expanded the classification into nine categories, incorporating systemic, metabolic, and developmental conditions [4,5,6].

The World Health Organization (WHO) [7] has introduced the International Classification of Impairments, Disabilities, and Handicaps, which further complements these frameworks by emphasizing the role of information and communication technologies (ICT) in enhancing the diverse needs of individuals with SHCNs and serves as a practical complement to traditional classification systems, offering solutions in accessibility, communication, and independence for individuals across sensory, physical, and behavioral domains.

The conditions mentioned above could significantly impact the oral health of children, which is essential for ensuring the well-being and overall health of individuals [2].

Extensive evidence suggests that SHCN individuals tend to experience significant disparities in oral health compared to those without such needs [8,9,10]. This includes a higher prevalence of untreated dental caries and periodontitis [10,11]. Furthermore, SHCN subjects commonly present with gingivitis, enamel hypoplasia, as well as orthodontic issues such as dental crowding and malocclusions, dental anomalies, bruxism and tooth wear; dental fractures or trauma are also frequently associated with SHCN individuals [2].

In pediatric patients with SHCNs, inadequate oral healthcare may stem from behavioral issues, such as dental anxiety and avoidance of dental settings, as well as challenges in performing oral hygiene maneuvers due to sensory difficulties related to toothpaste texture and taste, and the pressure exerted by toothbrushes [2,12,13].

Furthermore, oral health problems in pediatric individuals with SHCNs may be indirectly linked to the insufficient dental education of parents or caregivers and their apprehension about causing discomfort during oral hygiene practices for SHCN children [14,15,16]. This is compounded by the stigmatization and lack of support experienced by parents/caregivers from dental professionals, which further enhances the avoidance of dental care by both parents or caregivers and children [17,18,19]. Additionally, some dentists may lack knowledge in treating and managing the behavior of pediatric SHCN patients, overlooking their sensory profiles and failing to adapt the dental setting according to their requirements, such as addressing issues with bright lights or the noise generated by drills [20].

The use of digital technologies has become extremely popular today for bringing children closer to oral education culture, managing behavior during dental therapies, and increasing access to dental care, such as teledentistry, audiovisual distraction tools, and m-health apps [21,22,23].

To address the challenges often encountered by pediatric individuals with SHCNs, the emergence of digital technologies, such as m-health apps, has provided new avenues to promote adequate oral healthcare and to introduce SHCN children to the dental setting through education and familiarization. Indeed, dental m-health apps have the potential to address the shortcomings of traditional methods, which may not fully meet the specific needs of children with SHCNs. These apps could enhance oral hygiene skills, help children become familiar with the dental environment, and cost-effectively provide ongoing oral health education, even from home rather than solely during dental visits.

This narrative review aimed to examine available dental m-health applications designed for children with cognitive and physical impairments, exploring the perspectives of children, parents/caregivers, and dentists regarding their use and potential contribution to oral health. Based on these insights, a concept for a personalizable dental m-health app was proposed, considering the interactions between children, caregivers, and dentists to support oral care in pediatric SHCNs, with respect to the interconnectedness of the child, parent/caregiver, dentist, and medical specialists.

## 2. Materials and Methods

The Authors independently conducted an electronic search in MEDLINE/PubMed, Scopus, and Web of Science databases from 1 May 2024 to 14 September 2024, using the following keywords:

(“dental health” OR “oral health” OR “oral hygiene” OR “oral care”) AND (“m-health” OR “mobile health” OR “mobile app*” OR “smart toothbrush” OR “iPad” OR “tablet” OR “toothbrushing training” OR “wireless technology” OR “app-based” OR applications OR “visual aids” OR gamification OR “digital tools” OR “technology-based” OR “e-health” OR telemedicine) AND (children OR “pediatric” OR “adolescents” OR “young people”) AND (disability OR autism OR “autism spectrum disorder” OR “ASD” OR “physical impairment” OR “cognitive impairment” OR” Down syndrome” OR “special health care needs” OR “special needs” OR “neurodevelopmental disorders”) AND (“education” OR “training” OR “intervention” OR “program” OR “feasibility” OR “effectiveness” OR “behavioral support”)

An additional manual search was conducted to retrieve further relevant records.

This study included research published in English, with no restrictions on publication date, up to 14 September 2024. Eligible study designs encompassed a broad range of methodologies, including randomized controlled trials, cohort studies, case-control studies, cross-sectional studies, case series, case reports, and pilot studies. The study population focused on pediatric individuals with physical and cognitive impairments [3,4,5,6,7]. The interventions of interest involved the use of dental m-health applications designed to enhance oral health education, facilitate familiarization with dental care, support behavior management, or improve toothbrushing skills through interactive or passive engagement. The primary outcomes assessed were the reported effects of these digital interventions on oral health-related measures.

Conversely, studies were excluded if they were not published in English or if they fell under categories such as reviews, conference papers, commentaries, editorials, book chapters, or preclinical in vitro and in vivo research. Additionally, research involving adult populations or pediatric subjects without physical and cognitive impairments was not considered. Interventions that did not involve dental m-health applications or focused on non-dental m-health tools were also excluded, as were studies that failed to report relevant outcomes related to the impact of these interventions.

References were exported and managed through Mendeley Reference Manager Software, version 2.120.3, copyright Elsevier Ltd. (Amsterdam, The Netherlands).

## 3. Results

**Figure 1:**
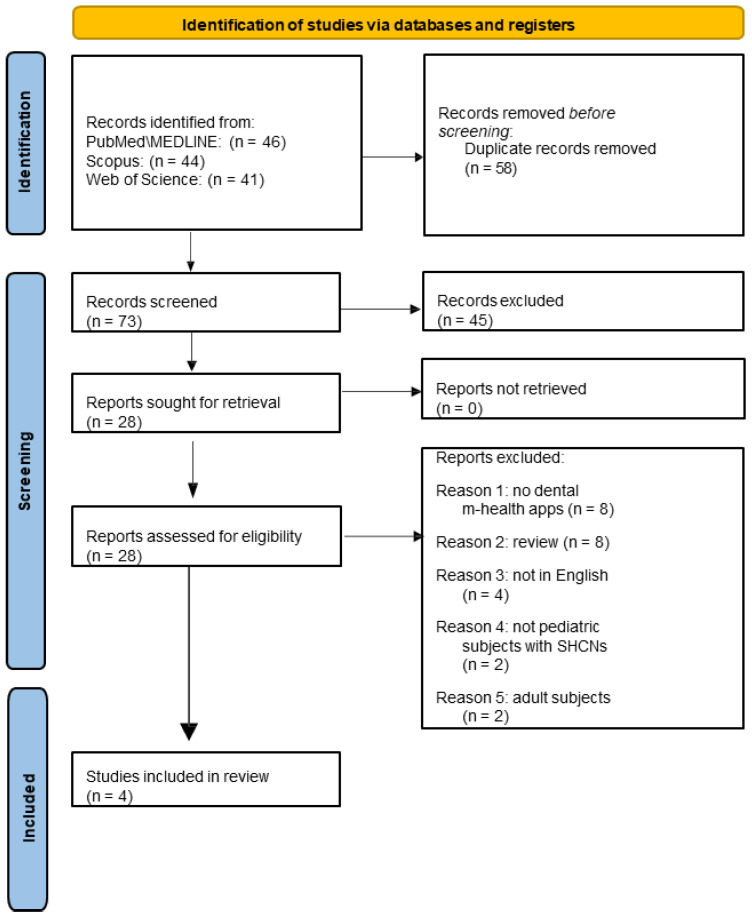
A flowchart of the study selection process of the electronic search.

The electronic search retrieved 131 records, including 46 from PubMed, 44 from Scopus, and 41 from Web of Science. The study selection process, illustrated in Figure 1, led to the inclusion of four articles [24,25,26,27].

After the electronic search, two additional articles [28,29] were included in this narrative review through a manual search.

Therefore, a total of six studies [24,25,26,27,28,29] were included in the present narrative review.

Most of the population involved in the included studies were diagnosed with ASD [24,25,26,28,29] and less frequently with Down syndrome [27]. The age of the population was 10.68 years on average and ranged from 4 to 16 years of age [24,25,26,27,28,29].

Some studies focused on using dental m-health apps to improve toothbrushing skills and oral hygiene motivation [24,25,29], while others were aimed at oral health education, familiarization, and behavior in the dental setting [26,27,28]. Specifically, two studies [25,27] evaluated the effectiveness of m-health apps alone, while two [28,29] combined the use of dental m-health apps with other techniques or tools—information and communication technologies (ICT) [28] and a smart toothbrush [29], respectively—one study [24] compared the use of the dental m-health app with conventional visual pedagogy, and one [26] evaluated the effectiveness of the dental m-health app for the oral health education of both caregivers and children [26].

Several studies [24,27,28,29] evaluated the use of dental m-health apps featuring interactive engagement, whereas others [25,26] focused on apps characterized by passive engagement.

### 3.1. Mobile Dental Applications Tailored for Children with Autism Spectrum Disorders

Most of the studies in the present narrative review focused on m-health apps designed for the pediatric population with ASD [24,25,26,28,29]. Specifically, five studies [24,25,26,28,29] evaluated dental m-health apps involving 173 children with ASD, with a mean age of 10.68 and an age range reported between 4 and 16 years.

The dental m-health apps targeted different goals: improving toothbrushing skills [24,25,29] and promoting oral health education, familiarization, and behavior management in the dental setting [26,28]. Among the 173 subjects with ASD, the apps were aimed at enhancing toothbrushing skills in 99 subjects (47.8% of the total population) [24,25,29], while oral education, familiarization, and behavior in the dental setting were addressed in 74 subjects (35.7% of the total population) [26,28].

In terms of intervention specifics, two studies [25,26] solely used dental m-health apps, while others combined the apps with additional technological tools or interventions [24,28,29]. One study [29] incorporated a smart toothbrush for 17 participants, another [28] paired the dental m-health app with an ICT program for 59 children, and one study [24] compared the app to conventional visual pedagogy in 30 children.

The reported intervention duration ranged from 28 to 240 days [24,25,26,28,29].

Two studies [24,29] reported the usage frequency of the dental m-health app, which was twice a day, with average session duration ranging from 1 min 58 s to 2 min 58 s.

Two studies [26,29] reported the usability ratings, which were negative in one study [29], due to technical issues in connecting the m-health app to the smart toothbrush and recording brushed areas, and positive in one [26], highlighting the ease of use and appealing design of the m-health app.

Dental m-health apps aimed at improving toothbrushing skills in pediatric subjects with ASD were effective in improving brushing quality and frequency [24,25,29].

One study [24] reported similar effectiveness of the apps compared with conventional visual pedagogy techniques.

Feedback reported on the child level was positive, as children described toothbrushing as more engaging and stimulating, believing the dental m-health app to be a tool for making oral hygiene routines more enjoyable and motivating [24,29]. In addition, pediatric subjects with ASD achieved greater autonomy in brushing, omitting fewer steps in the routine [24].

Feedback at the caregiver level indicated a positive opinion of using the dental m-health app, as it was helpful in tracking the child’s progress and encouraging adherence to instructions, making the oral hygiene-related routine less stressful [24,29].

One study [25] included feedback from dentists, who observed improvements in children’s independent completion of brushing steps and a reduction in refusal or complete failures over time.

Dental m-health apps aimed at oral health education, familiarization, and behavior control in the dental setting [26,28] in pediatric subjects with ASD were effective both alone [26] and in combination with the ICT program [28] in raising oral health awareness and care [26,28].

One study [28] reported feedback at the child level, which was positive, as the pediatric individuals improved their behavior and collaboration.

Feedback at the caregiver level indicated that dental m-health apps were positively welcomed and useful in oral health education [26,28].

One study [28] reported on dentist-level feedback, which was positive especially for the ability of the dental m-health app to be customized and adapted in ICT content to the needs of the child with ASD, increasing collaboration during dental treatments.

Table 1 shows the mobile applications tailored for children with autism spectrum disorders.

### 3.2. Mobile Dental Applications Tailored for Children with Down Syndrome

A single study [27] examined the use of a dental m-health app tailored for pediatric subjects with Down syndrome.

The study population amounted to 34 participants (16.4% of the total population), ranging in age from 7 to 12 years [27].

The intervention was based on the use of the dental m-health app to improve oral health knowledge and education [27].

The duration of the intervention was 7 days, with a usage frequency of once a day and an average session duration of 15 min [27].

The reported usability ratings were positive, as the dental m-health app was found to be easy to use. In addition, the use of the dental m-health app in pediatric individuals with Down syndrome was effective, improving oral health knowledge [27].

Moreover, at the child level, the dental m-health app was well received, engagingly enabling improved oral health education.

Caregivers also reported the app as user-friendly and engaging. No feedback was noted at the dentist level [27].

Table 2 shows the mobile dental applications tailored for children with Down syndrome.

### 3.3. Mobile Dental Applications Featuring Interactive Engagement

Four studies [24,27,28,29] evaluated the use of dental m-health apps featuring interactive engagement.

The population amounted to 140 pediatric subjects, of whom 106 had ASD [24,28,29] and 34 had Down syndrome [27], with a reported mean age of 10.67 and an age range from 4 to 16 years of age [24,27,28,29].

The dental care activity associated with app use included improved toothbrushing skills in 47 subjects with ASD (22.7% of the total population) [24,29], and an improvement in oral health education, familiarization, and behavior control in 93 subjects [27,28], of whom 59 had ASD (28.5% of the total population) [28] and 34 had Down syndrome (16.4% of the total population) [27].

The characteristics of a dental m-health app mentioned in one study [24], called “Brush Up”, with interactive engagement for improving toothbrushing skills, were based on an avatar showing the correct toothbrushing technique, while the child could observe themself brushing in the “selfie mirror” section [24].

The characteristics of the dental m-health app mentioned in one study [29] with interactive engagement were based on the connection between the m-health app and a smart toothbrush. The app generated an interactive gaming experience in which the child must fight virtual monsters in 16 areas of the mouth while the toothbrush records the brushing of the area [29].

Both apps aimed at improving toothbrushing skills [24,29] were based on positive reinforcement generated by virtual rewards or accessories to be unlocked by proper brushing while using the app. Both dental m-health apps were found to be effective in improving brushing skills and brushing frequency [24,29]. In addition, the apps’ interactivity and virtual visual rewards acted as promoters of proper oral hygiene [24,29].

The apps were found, on the children’s level, to be useful in increasing interest in dental routines and were well received.

On the caregiver level, the apps were valued for increasing independence in children and for the ability to track the progress achieved [24,29]. No feedback on the dentist level was reported [24,29].

The characteristics of a dental m-health app with interactive engagement for improving oral health education, familiarization, and behavior control, called “My Dentist”, were investigated by one study [28] as a combination of an app with ICT and a structured program to increase access to oral care, cooperation during treatments, and familiarization.

The app contained videos, social stories, personalized interactive games, and educational content that the dentist could customize to the specific needs of the child with ASD [28].

The use of the app was effective for children with ASD, as it improved access to dental care, children’s treatment behavior, and oral hygiene status [28].

On a child level, increased cooperation during treatments was found [28].

Caregivers had welcomed the app, and on the dentist level, it was reported that the ability to customize the app content improved children’s collaboration during dental treatment [28].

The characteristics of the dental m-health app mentioned in one study [27] included interactive engagement for improving oral health education, familiarization, and behavior control, called “Gigiku Sehat”, which encompassed animated videos and cartoons, interactive games for oral hygiene practice, and educational games and quizzes to test the knowledge learned through the use of the app, with positive reinforcement through virtual rewards [27].

The use of the app was effective in children with Down syndrome in improving oral health education and oral health promotion [27].

At the child level, the app was shown to be helpful in improving knowledge, and on the caregiver level, the app was found to be easy to use and engaging [27].

No feedback was reported at the dentist level [27].

### 3.4. Mobile Dental Applications Featuring Passive Engagement

Two studies [25,26] evaluated the use of dental m-health apps featuring passive engagement.

Both studies assessed the apps on a target population of 67 children with ASD, with a reported mean age of 10.2 years [25] and an age range of 3 to 12 years of age [26].

The dental care activity associated with app use improved toothbrushing skills in 52 subjects (25.1% of the total population) [25], and improved oral health education, familiarization, and behavior control in caregivers and children in 15 subjects (7.2% of the total population) [26].

The characteristics of the dental m-health app, called the “çATED” app, with passive engagement for improving toothbrushing skills [25] were based on uploading photos or pictograms to divide the steps of the toothbrushing routine into 12 or 16 steps, such that they would be more followable by a child with ASD. The dentist evaluated brushing at baseline, after 4 months, and after 8 months by assigning a score for each step shown in the app, indicating whether or not the child performed it and whether the child performed it independently or was assisted by a caregiver.

The passive engagement dental app “çATED” for toothbrushing skill improvement [25] was effective, and increased autonomy was found on the child level. At the dentist level, an improvement in toothbrushing skills was observed. Over time, the total failures across all steps of the toothbrushing process decreased, and fewer steps in the brushing routine were omitted [25].

However, no child was able to complete all the steps independently without assistance [25].

No caregiver-level feedback was reported [25].

The characteristics of the dental m-health app with passive engagement aimed at oral health education, familiarization, and behavior control, called “Nus Care” [26], was based on the use of social stories set in the dental environment (such as visiting the dentist, taking X-rays), visual schedules and educational contents (foods that likely cause tooth decay, photographic examples of dental traumas), rewards (after accomplishing activities such as brushing teeth) and a calendar (for appointments reminders) for both children with ASD and their caregivers/parents [26].

**Figure 2:**
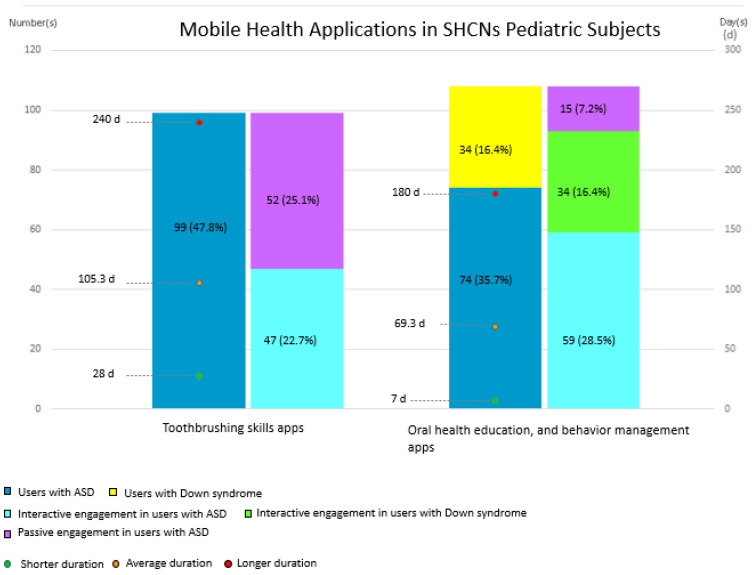
Mobile dental applications for children with cognitive and physical impairments, categorized by the app’s goal (toothbrushing skills on the left, oral education and dental behavior management on the right), by engagement (interactive/passive), and by the type of target population (ASD, Down syndrome). The reported duration of the intervention applied to the population is expressed in days (d), with shorter, longer, and average durations indicated. The number of users is reported, with the percentage relative to the total study population in brackets.

Figure 2 shows the mobile dental applications for children with cognitive and physical impairments.

## 4. Discussion

Oral care is an unmet healthcare need that poses significant challenges, especially in the context of children with cognitive and physical impairments [2,30]. Indeed, these children tend to experience poorer oral health compared to peers without impairments, likely due to particular sensory profiles that may make it difficult to perform oral hygiene maneuvers [2]. These profiles may include problems related to sensory stimuli such as toothpaste textures, tastes, or toothbrush pressure, as well as behaviors that lead to avoidant attitudes toward new environments, contributing to dental fear and anxiety [2]. Considering that oral health is closely linked to an individual’s overall well-being and has a significant impact on quality of life [31], it is critical to reduce the disparities that this population often experiences [8,9,10].

The use of modern technologies such as teledentistry, audiovisual distraction tools, and m-health apps has emerged as a strategy to improve oral care and dental education in pediatric SHCN patients [32,33].

Therefore, the present narrative review aimed to examine available dental m-health applications designed for children with cognitive and physical impairments, exploring the perspectives of children, parents/caregivers, and dentists regarding their use and potential contribution to oral health. Based on these insights, a concept for a personalizable dental m-health app was proposed, considering the interactions between children, caregivers, and dentists to support oral care in pediatric SHCNs, in light of the interconnectedness of the child, parent/caregiver, dentist, and medical specialists.

### 4.1. Children and Caregivers’ Perspectives

Based on the findings of the present review, from the children’s perspective, dental m-health apps have been effective in improving toothbrushing skills and implementing oral health education, familiarization, and behavior control.

Pediatric individuals with SHCNs experience numerous difficulties in establishing and maintaining proper oral care habits [34]. These issues might relate to communication and behavioral challenges and altered sensitivity to textures, tastes, pressures, or noises associated with dental hygiene practices. Such difficulties frequently lead to a lack of consistency in daily home oral hygiene maneuvers and an avoidant attitude toward the dental setting [35].

Evidence suggests that the oral care of these children is mainly dependent on caregivers, who often report feelings of frustration and difficulty in managing hygiene routines [36,37]. This situation contributes to limited accessibility to oral health, both at home and in the dental office [35]. As a result, children with SHCNs have a significant disparity in oral health compared to peers without such needs [10,11].

Therefore, it may be essential to understand the perceptions and experiences of children to enable them to participate actively in activities related to their oral health. Indeed, tailored interventions designed to meet the specific needs of these patients might foster acceptance and engagement while promoting self-determination and autonomy. A personalized approach may not only improve adherence to oral hygiene practices but also contribute to the child’s overall well-being, reducing stress on caregivers and facilitating positive integration with the dental setting [37,38].

Various interventions aimed at improving toothbrushing skills have been developed to address disparities related to inadequate oral hygiene in children with SHCNs. These include visual pedagogy, audiovisual aids, and books and videos with social stories or picture exchange communication systems (PECS), which are particularly effective in pediatric patients with ASD [35,39,40].

The use of new digital technologies, such as m-health applications, might significantly enhance home oral hygiene management in children with SHCNs by providing an innovative and personalized approach that facilitates learning and practicing oral hygiene maneuvers.

Indeed, in the present narrative review, children reported that the brushing experience was more engaging and enjoyable through the use of dental m-health apps, whether they involved interactive or passive engagement by the user. Most of the interactive apps examined were based on game experiences that allowed the child to view an avatar or that allowed the child to defeat monsters through brushing. It is plausible to assume that the child’s engagement and participation were ensured by involvement through play and the possibility of receiving virtual rewards by advancing their level of expertise [41]. In non-SHCN children, the use of interactive games improved oral hygiene knowledge and habits, with improved clinical parameters [42].

Although clinical parameters were heterogeneously investigated in the present narrative review, the interventions based on dental m-health applications effectively improved oral health. One study [24] reported a reduction in plaque index in children with ASD, from 2.01 (±0.06) at baseline to 1.00 (±0.00) at six weeks, with a further decrease to 0.46 (±0.11) at 12 weeks. Similarly, the gingival index decreased from 1.03 (±0.17) at baseline to 0.58 (±0.16) at 6 weeks and to 0.24 (±0.11) at 12 weeks, with no significant differences from the visual pedagogy-treated group, suggesting that both methodologies are equally effective. While specific measurements to evaluate brushing performance improvements were not employed, other studies [25,29] indicated enhanced brushing activity, increased brushing passes performed independently by children, and more comprehensive coverage of tooth surfaces, as well as improved brushing skill quality, frequency, and duration. In addition, dental m-health applications have shown a reduction in anxiety-related parameters, both through self-reported measurements and through objective assessments, such as electrocardiogram and galvanic skin response, highlighting the potential of these tools in fostering familiarization with the dental environment and contributing to the reduction of dental fear and anxiety associated with dental care.

In addition to interactive dental m-health, apps with passive engagement have also been shown to implement brushing skills effectively. In fact, for some SHCN children, it might be particularly beneficial to break down a complex task, such as brushing, into several sub-tasks through steps drawn or depicted as pictures [43]. Although, in some cases, the children still needed caregiver assistance during brushing, the dental m-health apps allowed them to perform more steps independently.

In this narrative review, a relevant observation was that children with Down syndrome tended to more frequently use dental m-health applications characterized by interactive engagement. In contrast, children with ASD engaged with a more varied range of approaches, which included both passive and interactive features. This difference could reflect the specific needs of the two populations but might also be attributable to the limited diversification in application development rather than a real preference or effectiveness of one approach over the other. In addition, it should be considered that more of the included studies focused on children with ASD, which may have influenced this distribution.

Another notable consideration is the duration of the interventions: applications aimed at improving brushing skills tended to be used for longer periods than those oriented toward oral health education and behavior management.

This finding could indicate that consolidating brushing skills requires repeated and prolonged training. Unlike educational applications, which focus primarily on information transmission, improving brushing skills also involves the connection between perception, movement, fine motor control, hand–eye coordination, movement planning, and the repetition required to automate the gesture [44,45].

In children with SHCNs, these skills may take longer to consolidate, which may explain the longer duration of interventions focused on this area.

These findings highlight the need to expand research to other categories of individuals with cognitive and motor disabilities, evaluate the effectiveness of interactive versus passive approaches more broadly, and further investigate the duration of interventions in different subpopulations.

Moreover, according to the findings of the present review, dental m-health apps that implement oral health education, familiarization, and behavior control have demonstrated effectiveness in improving the education of both children and their caregivers.

Caregivers reported positive feedback regarding the acceptability and usability of the m-health apps, considering them valuable tools for increasing knowledge of the importance of oral health, improving familiarization with dental practices, and managing behavior.

Thus, caregivers of children with SHCNs frequently face high stress because of the child’s behavioral difficulties, especially in settings such as dental care, where the new environment can increase frustration [46,47]. Caregivers’ social isolation, amplified by stigma, reduces their access to dental care and limits their knowledge about oral hygiene practices [17,19]. Parents often fear causing harm during oral hygiene maneuvers, especially if the children cannot perform these practices independently [14,48]. This fear can generate anxiety and frustration, leading to neglect in the regular practice of oral hygiene [49]. Consequently, a lack of confidence in adequately managing these practices might contribute to oral health problems. Parents should receive proper education to help them overcome these fears and confidently perform the maneuvers, ensuring regular and proper oral care for their children. Psychological and educational support by dentists might be essential in reducing parental anxiety by helping caregivers tailor management strategies for SHCN children’s needs and improving the quality of oral care to promote good dental health in the long term.

Therefore, to improve care, it is crucial to develop educational resources not only for children but also for caregivers so that they can better support their children. The relationship between the child, caregiver, and dentist should be viewed as an interconnected circuit directly affecting the child’s oral health. Positive interaction among all participants in the caregiving process is essential to promote change in oral care habits and reduce inequalities in access to care.

Some studies suggest that using audiovisual tools, such as tablets and smartphones, during dental visits helps manage the child’s anxiety and behavior, facilitating a less stressful dental experience [50,51]. In addition, teledentistry, which offers remote support, has positively impacted equitable access to care, especially for caregivers with socioeconomic difficulties or who live in areas with few specialists [52]. The use of these tools helps not only to improve the child’s oral health but also contributes to the improvement of the caregiver’s oral health by increasing awareness and encouraging the adoption of proper habits [53,54]. The use of dental m-health apps may further improve the quality of care and reduce inequalities by providing long-term support that continues in the home setting.

**Figure 3:**
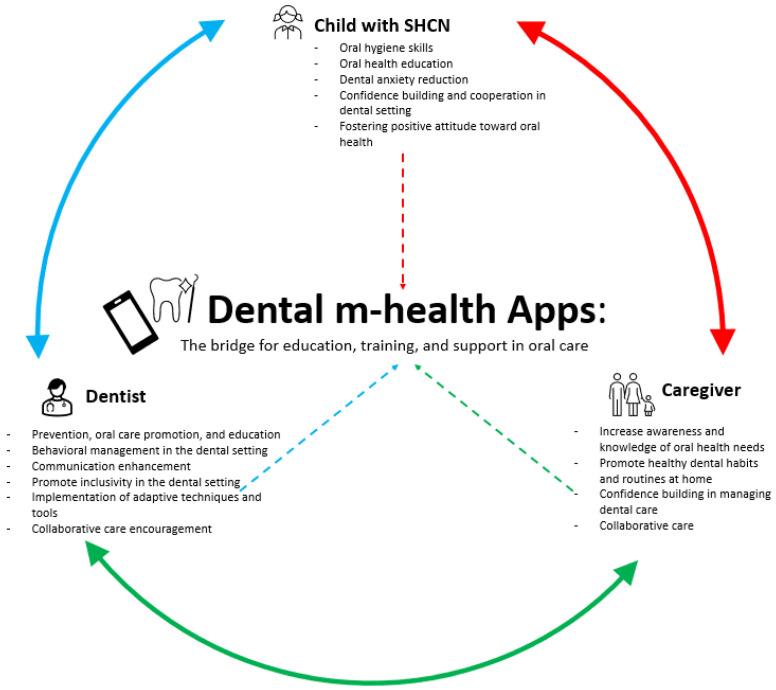
The potential of dental m-health apps in the child, caregiver, and dentist interconnection.

Figure 3 shows the potential of dental m-health apps in the interconnectedness of the child, caregiver, and dentist.

However, although the present study highlighted the potential usefulness and effectiveness of dental m-health apps, it should be considered that they have been used on limited categories of impairments, such as ASD and those with Down syndrome, but not on other needs in the SHCN population, such as those with hearing or vision impairments. Larger studies may be needed to analyze the use of dental m-health apps in the pediatric population with SHCNs and the relative perceptions of children and caregivers.

Dental m-health apps may represent an innovative resource for reducing costs in the national healthcare system, especially in pediatric care for children with SHCNs, who often require complex care and have higher care costs than patients without SHCNs [55].

Dental m-health apps may reduce healthcare costs for children with SHCNs through features such as oral health education, remote monitoring, behavioral support, and dental emergency prevention. These tools could prevent costly conditions, reduce frequent visits, and lower costs associated with sedation and hospitalizations, positively impacting the national healthcare system by improving effectiveness and access to care in an economically sustainable way.

### 4.2. Dentists’ Perspective

In the present narrative review, although reported dentist-level feedback was limited, the use of m-health apps, especially when tailored, has proven effective in increasing child autonomy in oral hygiene routines and collaboration during treatment [25,28].

Dentists play a direct role in providing care for pediatric subjects with SHCNs and in making dental setting-related experiences positive [1]. SHCN subjects tend to have a higher prevalence of untreated dental conditions than the non-SHCN population [10,11].

Indeed, SHCN subjects tend to have a higher prevalence of untreated dental diseases such as caries or periodontitis than the non-SHCN population [11,56]. This could be due to the dental fear or anxiety that pediatric SHCN subjects sometimes possess due to sensory sensitivities, overstimulation from the environment, and fear of the unfamiliar, which sometimes manifest in aggressive and uncooperative behaviors [57] resulting in an over-reliance on general anesthesia in dentistry [38,58].

However, the existing disparity in oral care compared with the non-SHCN population could result from dentists’ lack of knowledge about the management of pediatric SHCN subjects and the stigma that may sometimes be present [18,59]. Since oral health is an essential component of overall well-being [2], dentists should have adequate training and experience such that they can manage behavior, adapt the dental environment to sensory needs and profiles, and promote oral health literacy and prevention in children with SHCNs [60,61]. Several interventions showed potential in bringing people closer to oral health education, such as animated videos, peer modeling, or social stories [62,63].

Thus, to support the dentist in the behavioral management of SHCN children in the dental setting, alongside traditional behavioral management methods, a growing interest has developed in digital technologies such as audiovisual distraction techniques, for example, video or cartoon projections or support with technological tools such as visors or goggles for virtual or augmented reality [64,65]. Specifically, for the management of the SHCN child in the dental setting, it has been proposed to combine the use of traditional behavioral management techniques such as tell–show–do with audiovisual distraction tools and, through short, time-delayed visits, familiarize the child with the dental setting [66].

Although these interventions can help manage dental treatments or visits, one aspect that should be considered is the importance of prevention and promotion of proper oral hygiene by dentists [61]. Indeed, SHCN children tend to have to receive more invasive dental treatments than the general population, likely due to difficulties in maintaining oral hygiene routines, sensory challenges, or lack of awareness in families, and inadequate dietary habits, which can contribute to the development of oral health issues [19,36,67].

Educating families on proper nutrition and oral hygiene practices might, therefore, be essential in reducing the risk of dental complications in this population. From this perspective, dental m-health apps may be a valuable tool to enhance oral hygiene activities at home and spread more knowledge about the importance of oral health, the role of nutrition, regular visits, and home and professional oral hygiene.

Although the findings of the present narrative review were limited, the potential effectiveness of the reported dental m-health apps may limit oral health disparities in the SHCN population. Further studies and a broader study population are necessary to assess the effectiveness of dental m-health apps in SHCN pediatric subjects.

### 4.3. Proposal of a Tailored Application

The findings of the present narrative review highlighted the paucity of resources available to the pediatric population with SHCNs. Individuals with SHCNs face numerous social, individual, professional, and governmental barriers to accessing appropriate care and interventions [68]. These difficulties are related to structural and organizational limitations of healthcare services, as well as to stigmatization and a lack of specific training [14].

Indeed, children with SHCNs face a heightened burden of oral health challenges stemming from both individual, systemic, and socioeconomic factors [2,8,10]. Conditions such as dental caries, periodontal disease, enamel erosion, and malocclusion are particularly prevalent in this population [1,41]. For instance, studies report higher caries prevalence rates in children with ASD, attributed likely to restricted dietary preferences, oral sensory sensitivities, and medication-induced xerostomia [1,67,69]. Similarly, children with Down syndrome exhibit high periodontal disease rates, influenced by immune system impairments, open-mouth posture, and poor plaque control [69,70,71]. Children with cerebral palsy commonly experience enamel erosion due to gastroesophageal reflux [69,72]. Additionally, children with cognitive and intellectual impairments face difficulties in adaptive behavior, such as toothbrushing and hygiene management, which leads to higher plaque accumulation and a significantly worse gingival status compared to typically developing peers, often progressing to periodontitis [69,72].

Globally, the burden of disabilities is unevenly distributed, with an estimated 240 million children worldwide living with disabilities [73].

In some regions, dental care systems emphasize immediate needs over preventive measures and specialized services, while fragmented and poorly integrated healthcare systems may further restrict access to necessary care [74].

In contrast, high-income regions benefit from universal or hybrid healthcare models, which enable better integration of oral health into general healthcare policies [74,75,76]. However, challenges persist even in these regions, likely due to workforce shortages, limited provider training, and gaps in service delivery for children with SHCNs. Public programs provide essential dental coverage for pediatric subjects with SHCNs [70,77]. However, these services often face barriers, including low reimbursement rates, which might discourage provider participation, limiting access to care [75,77]. Consequently, the majority of dental care is primarily delivered through the private sector [77].

Although legislative frameworks promote equitable access to healthcare for individuals with disabilities, regional disparities in resource allocation and service availability might still persist, highlighting the need for targeted strategies and innovative solutions to bridge these gaps.

Despite these challenges, advances in technology have made health information more accessible and have shown promise in addressing care gaps, even in underserved areas [78,79].

The present review highlighted the increasing potential of dental m-health applications for pediatric patients with SHCNs, suggesting that these technologies can be valuable tools for improving accessibility to dental care and toothbrushing skills and promoting oral health education.

However, the review highlighted a limited number of applications specifically targeting this population, with a primary focus on children with ASD and Down syndrome.

The findings of the present narrative review underscored the need to develop inclusive and innovative tools tailored to the specific needs of these children, particularly in the Italian context, where regional disparities in care delivery remain a challenge.

In light of these considerations, the Authors proposed an innovative, tailored dental m-health app designed for pediatric SHCN patients, emphasizing a comprehensive approach involving children and caregivers, dentists, and medical specialists. Existing applications for this population often focus primarily on children and, in some cases, their caregivers, while the direct involvement of dental professionals remains limited. Findings from this narrative review, including the study by Narzisi et al. [28], suggested that integrating dentist- and child-centered features within a customized platform could be effective, particularly for children with ASD. Building on this evidence, the proposed app features two distinct interfaces at login—one for parents/caregivers and children and another for dentists and medical specialists—ensuring a personalized and integrated approach to dental care. Moreover, the app is structured according to the principles of P4 medicine: Predictive, Preventive, Personalized, and Participatory [80,81].

Specifically, these can be elaborated as follows:

− Personalization: The app can be customized both by the child to fit individual patient needs through the ability to choose specific content, the interaction mode, and app graphics and colors, and by the dentist in dentist mode, as they can include content specific to their patient and tailored to their needs (e.g., if the child is to receive conservative treatment, the dentist can upload videos and information related to the treatment so that the child knows what to expect and can become familiar with the sounds of the instruments and the treatment they will be undergoing, with opportunities for increased collaboration.
− Prevention: The app includes features for monitoring patient progress and educational content for children and parents, such as content on the role of nutrition in the prevention of dental caries.
− Prediction: The app collects progress data with app interaction. These data allow the dentist to personalize content for children progressively with new topics, so as to increase dental education over time.
− Participation: The app could encourage patients’ active participation in their own care process by allowing them to participate in interactive activities that improve skills—such as educational games on treatment management, videos, and interactive toothbrushing skills content—increasing their autonomy during oral hygiene routines. In addition, the application also allows for the active participation of caregivers/parents, dentists, and medical specialists.

This approach allows for coordinated linkage and support among all participants involved in the oral health of the SHCN subject: child, parent/caregiver, dentist, and medical specialists. The goal is to promote comprehensive oral health support capable of adapting to most sensory profiles of pediatric SHCN subjects by improving cooperation, facilitating behavioral control during dental treatments, and overall oral health education.

The integration of these features into the dental app proposal is based on the need to create an inclusive and personalized m-health application that adapts to the needs of the majority of pediatric SHCN categories by also involving parents/caregivers and dentists along with medical specialists, facilitating a comprehensive approach to oral health. In addition, the proposed dental m-health app, based on the findings of this narrative review, where both passive and interactive m-health apps have shown effectiveness, is designed to include both passive and interactive features.

This strategy aims to maximize the potential benefits and to further adapt to the needs of all parties interacting with the proposed dental m-health app. Most m-health apps in the present review showed limitations, as they often targeted a single pediatric SHCN group and may not have accommodated or provided sufficient adaptations for children with different sensory profiles and needs.

In addition, such apps are predominantly targeted at children, excluding the active involvement of parents and health professionals, who are key elements of integrated oral health management. Therefore, the Authors’ proposal for a tailored, multi-user dental m-health app is based on the following main goals:

− Adaptability for different sensory profiles: customizable functions to meet each child’s specific needs.
− Multi-user: differentiated access for child/caregiver and dentist.
− Increased oral care and prevention: dedicated oral hygiene sections and animated multimedia content.
− Familiarization with the dental environment: multimedia content to reduce anxiety and fear.
− Knowledge and awareness for caregivers: educational sections to increase knowledge and support.
− Communication interface: the ability for caregivers to interface with the dentist and feel supported in caring for the SHCN child.
− Visual monitoring: camera function to take intraoral photos and send them to the dentist.
− Search for specialists: option to locate specialists in the area.

The proposed design of the dental m-health app is based on adherence to the objectives and the integration of what was inferred from this narrative review.

As described above, the app has two interfaces: child/caregiver and dentist/medical specialist, which share the same home consisting of the following sections:

(a) Settings;
(b) Profile;
(c) Camera;
(d) Calendar;
(e) Let’s Brush Teeth Together!;
(f) Video guides;
(g) Book a visit;
(h) Find your specialist;
(i) Tips and questions.

#### 4.3.1. Child and Caregiver Mode

The design of this interface mode, through access to the app in the mode of “I am a parent/child”, aims at integrated support and dissemination of knowledge and oral health prevention.

In the “Settings” section, the caregiver, considering the specific needs and sensory profiles of the SHCN child, will be able to customize the application through the choice of the colors of the background layout; the choice of the use of click or verbal command; the presence or absence of alternative text or subtitles; the presence of beeps or vibrations while using the section dedicated to oral hygiene; the choice of background music loaded from one’s music library; the activation or deactivation of push notifications; and the choice of an avatar.

In the “Profile” section, the parent will be able to view the child’s progression over time, upload intraoral photos, and monitor adherence to daily and weekly oral hygiene goals. The caregiver will be allowed to access a sub-section containing “notes for the dentist”, in which the child’s sensory profile characteristics could be selected, and a small report on previous dental experiences and a subsequent evaluation of the child’s behavior could be completed, all by selecting items from a drop-down menu. For example:

− My child doesn’t like… (Selection: bright light, noise, physical contact…)
− Has the child been to the dentist yet? (Selection: yes/no.)
− If yes, for what reason? (Selection: visit, cavities, or extractions.)
− How do I rate the dental experience concerning the child’s behavior? (Selection: positive experience, moderately positive experience, moderately negative experience, or negative experience.)
− How was the child managed? (Selection: no need for particular techniques, musical or audiovisual distraction techniques, sedation, or general anesthesia).

This section aims to curb—in relation to what the evidence suggests—stigmatization that is often perceived by parents, thus increasing parental participation and making them take a specific interest in their child. It also aims to provide the dentist with a preliminary picture that can guide dental care toward behavioral and dental environment management that is appropriate to the needs of the SHCN child.

The “camera” section allows for taking intraoral photos of the child. The caregiver will be able to monitor the child’s oral hygiene status, as the app, through artificial intelligence, can categorize the photos into mild, moderate, or severe plaque accumulation. This section also allows interfacing with the dental specialist, as by taking intraoral photos, the dentist can actively support the parent’s concerns.

Indeed, children with SHCNs may find it particularly difficult to adjust to new environments, such as the dental office setting. Allowing parents to consult the child’s specialist directly from home via intraoral photos may be a less stressful solution for the young patient. In this way, parents can receive support and clarification of their concerns, while the child can gradually become accustomed to the idea of an in-person dental visit.

The “agenda” section will allow the child/caregiver to see appointments with their specialist and set daily goals.

The “Let’s Brush Together!” section aims to support the SHCN child in daily oral hygiene maneuvers.

The section will have the same order of brushing of quadrants, signaled by an acoustic or written signal on the screen, since it has been shown that pediatric SHCN subjects, compared to non-SHCN subjects, are prone to forming habits as long as they are of a stereotypical order [82].

This section has brushing reminders according to the times entered by the caregiver and can record the duration of use, frequency, and average time of the sections with daily and weekly reports.

The “Video Guides” section contains video tutorials to educate the child on oral hygiene maneuvers and the ability to view content on the dental treatments to be performed to minimize anxiety or fear related to the unknown. In addition, this section allows the child to familiarize themself with the dental environment, as the dentist can upload pictures or videos of their office rooms or waiting room.

The “Book a Visit” section will allow people to book appointments with the dentist and receive reminders in advance.

“Find Your Specialist” was created to generate a multidisciplinary and interdisciplinary collaboration between dentists and other medical specialists to provide the most comprehensive dental care possible for the pediatric SHCN subject.

In addition, parents/caregivers can find a nearby specialist to ensure as many possibilities as possible for accessing care.

#### 4.3.2. Dentist Mode

The dentist mode covers the same sections as the child/caregiver mode described above. The intention is to maintain the same screen so that parents can see the app’s functionality during visits, take children to access care, and view their profiles.

During the in-person visit, the dentist can use the camera section to upload the child’s profile, follow up on the oral hygiene status, and monitor this status even from a distance. In addition, the dentist can upload personalized content in the video guide section, customized for different children to the treatment they will be undergoing, as well as photos of the dental staff and environments to familiarize the SHCN child.

Moreover, the proposed dental m-health app facilitates an interdisciplinary approach to treating children with SHCNs by integrating different dental specialists. For instance, conservative dentists may monitor and diagnose carious lesions early through telemonitoring, while endodontists may telediagnose based on reports and images shared by parents. Other specialists, such as periodontists and orthodontists, may collaborate in tailoring treatments, ensuring targeted and continuous intervention by sharing updated information on the patient’s progress and tailoring the treatment plan to the child’s specific needs.

In this way, the app may facilitate continuous monitoring and communication between the dental team and the family, providing accessible and timely specialist support tailored to the patient’s individual needs and parental/caregiver concerns.

This system could improve access to care by providing personalized and immediate management of the child’s dental needs.

### 4.4. What Does the Future Hold?

The Authors’ proposal for the dental m-health app attempts to facilitate interaction among all components of the child’s relational networks, ensuring the dissemination and education of oral health through a multimodal approach.

First and foremost, it is aimed at pediatric SHCN individuals to enhance their oral hygiene skills while increasing their autonomy.

Moreover, it could help mitigate adverse behaviors in children which are often associated with the dental environment, such as dental anxiety and fear. By leveraging animated multimedia content or peer modeling videos, pediatric SHCN patients can progressively familiarize themselves with the dental environment, ultimately improving compliance during visits, treatment acceptance, and overall care experiences.

The individual can also be prepared for upcoming dental visits by introducing them preliminarily to the staff and the dental office setting, including the waiting room, to minimize anxiety related to the unknown and unfamiliar environments, which often prevail in children with SHCNs.

Furthermore, step-by-step tutorial videos will guide them through treatment procedures, fostering a sense of predictability and reassurance. Gaining this awareness through digital learning may particularly benefit children with SHCNs, who often struggle to adapt to new experiences.

Additionally, the model is designed to provide active support to the caregivers/parents, who can interact with the dentist and describe, through a questionnaire, the child’s sensory and communication-related difficulties and previous dental experiences.

Additionally, parents will have the opportunity to receive timely feedback on their child’s oral health status through photos. They will also be able to consult local specialists to ensure comprehensive and specific care tailored to the child’s needs.

The dentist, in turn, can actively support both the parent and the child with SHCNs by fostering a collaborative and relaxed atmosphere during treatment and promoting oral health. Furthermore, the dentist can collaborate and interface with other specialists, creating a strong network of relationships that can overcome and address the challenges faced by the SHCN population and aim to improve health outcomes.

Notably, the present review examined a limited range of SHCN conditions. Therefore, future research should consider how different sensory profiles among these children may influence the effectiveness of dental m-health apps. The clinical implementation of the proposed app could provide valuable insights into the perception of different SHCN subgroups and the response to such interventions to optimize tailored digital solutions for this population.

The proposed dental m-health app has been conceived in Italian, but future development may include an English version to achieve a broader reach.

In the future, the conceptualized app could be introduced to the pediatric SHCN population to evaluate its effects, the acceptance of the app by children, parents, and dentists, and the adherence to the goals that it sets.

Future studies involving children with special healthcare needs, their caregivers, and dentists can achieve clinical validation of the application. These studies will allow the evaluation of the interface’s effectiveness and acceptability in different subgroups of users, gathering feedback from children, parents, and professionals for possible improvements to the prototype. Moreover, a specific questionnaire for parents/caregivers could assess their perception of their children’s oral hygiene status, the app’s usability and acceptability, and its perceived impact on daily routines.

The app’s impact on oral health could be measured over time through the assessment of clinical parameters, such as plaque index (PI) and bleeding on probing (BOP), continuous monitoring through intraoral photos provided in the app, and assessment of anxiety and dental behavior through self-reported questionnaires and objective measurements. Furthermore, the incidence of carious lesions over time could be monitored through the app to evaluate the long-term effectiveness of the app on oral health. The improvement of dental awareness among children and their caregivers will also be analyzed.

The handling and storage of personal data for the app’s implementation will require strict consideration of ethical and legal aspects, under the General Data Protection Regulation (GDPR) of the European Union. It will also require obtaining explicit and verifiable informed consent from parents or legal guardians to collect personal data from minors, ensuring maximum privacy protection and security of the information collected.

Future perspectives for the proposed dental application include integrating advanced AI features or other smart technological tools to personalize content further based on the child’s progress and preferences, enabling more tailored interventions and accurate monitoring.

There is also the potential for expanding the app’s functionality to include real-time communication features between parents and specialists, allowing for more immediate guidance and support. Furthermore, the app could evolve into a comprehensive health management tool, encompassing dental care and other areas of health that affect children with SHCNs, promoting a more comprehensive approach to their well-being. Long-term goals may include creating a broader network for data collection and research on the effectiveness of digital health tools in pediatric SHCN care and facilitating the development of evidence-based practices.

In the future, m-health apps could also be used to train dental students and provide resource support for the pediatric special-needs population.

In the future, these apps may offer interactive educational resources, clinical simulations, and real-world case studies that enable dental students to acquire practical and theoretical skills.

These resources can fill the knowledge and information gap that many dentists have regarding the care of the pediatric special-needs population. Traditional training programs often do not adequately address these fields.

These apps could improve students’ awareness, preparedness, and competence in treating patients with complex and diverse needs.

### 4.5. Strengths and Limitations

One limitation of the present study, being a narrative review, is the absence of a pre-established systematic approach, which could lead to potential biases due to the differences in population characteristics, study designs, and methodologies among the studies included. Additionally, the limited number of available studies and the lack of data on the long-term contribution of dental m-health apps in oral health may represent further limitations that should be addressed. Moreover, the heterogeneity in methodology and diagnostic criteria among the included studies may have influenced the interpretation of the findings, underscoring the need for more standardized research approaches in this field.

Nevertheless, narrative reviews offer perspectives on a topic and contribute to expanding knowledge and awareness. Indeed, the present review aimed to explore the use of dental mobile health apps for pediatric patients with physical and cognitive impairments and the perceptions held by children, caregivers, and dentists. Despite some limitations, this narrative review offers insights into how dental m-health apps can enhance brushing skills, provide oral health education, and help familiarize children with dental procedures. It also examined how these apps’ interactive and passive features engaged children.

In addition, to the best of our knowledge, this review provides, for the first time, a proposal for a new dental m-health app capable of adapting to the majority of specific needs and which considers child, caregiver/parent, dentist, and medical specialists. The proposed app is a conceptual idea based on the current literature gaps and has not yet been developed.

## 5. Conclusions

The present narrative review highlighted the potential of dental m-health apps as valuable tools for enhancing brushing skills and oral health education, familiarization, and behavior in pediatric subjects with physical and cognitive impairments.

The effectiveness of these digital tools, both through passive or interactive engagement features, suggested a promising role in promoting oral health and facilitating patient cooperation.

In addition to improving oral health behaviors, these digital solutions could significantly help reduce healthcare disparities by enhancing access to preventive care for children with special healthcare needs, particularly those who encounter barriers to traditional dental visits. Integrating dental m-health apps into routine pediatric dental care may overcome gaps in oral health education and professional guidance, potentially fostering better long-term oral health outcomes.

However, the limited availability of dental m-health apps specifically targeting pediatric patients with SHCNs reinforced the need to invest in research, development, and clinical validation of these tools.

Future research should focus on large-scale trials, involve a broader population to investigate the integration of these technologies into clinical practice, evaluate their impact on long-term oral health outcomes, and explore strategies to strengthen collaboration among children, caregivers, and dental professionals.

## Figures and Tables

**Table 1:** Characteristics of mobile applications tailored for children with autism spectrum disorders: population, methods, intervention, engagement and usability, outcomes, feedback, and sustainability.

Study	Population	Methods	Intervention	Engagement and Usability	Outcome(s), Feedback, and Sustainability
France 2024 Children [29]	Nr. of participants: 17 Mean age: 8.5 y.o. (age range 5–12 y.o.) Target population: ASD	Aim: improving toothbrushing motivation and effectiveness through use of a smart toothbrush associated with app	Intervention: smart toothbrush and app Dental care activities: interactive toothbrushing App characteristics: interactive game for toothbrushing, virtual rewards for proper brushing Duration: 28 days	Engagement: interactive Usage frequency: twice a day Average session duration: a mean of 1 min 58 s Usability ratings: technical issues related to connecting the app with the smart toothbrush and recording the areas brushed (self-reported)	Main result: the use of smart brush and app was effective in improving brushing frequency and quality. Child level: increasing interest and motivation in brushing, stimulated by the game and positive reinforcement. Caregiver level: increased independence and reduced stress during oral hygiene routine. Dentist level: MD.
Krishnan 2021 Spec Care Dentist. [24]	Nr. of participants: 30 Mean age: 14.29 y.o. (age range 14–17 y.o.) Target population: ASD	Aim: evaluate and compare use of visual pedagogy with m-app for brushing education and promotion of good oral health status	Intervention: “Brush Up” app Dental care activities: interactive toothbrushing App characteristics: brushing is guided by an avatar showing the correct technique while the child can observe themself brushing; virtual rewards for proper brushing Duration: 84 days	Engagement: interactive Usage frequency: twice a day Average session duration: 2.75 min Usability ratings: MD	Main result: the app has proven effective in promoting good oral hygiene by taking advantage of interactivity and visual reward. The app has a similar effectiveness to visual pedagogy. Child level: the app was well received, increasing engagement and motivation in the toothbrushing routine. Caregiver level: the app was supportive and allowed them to track progress and encourage adherence to instructions. Dentist level: MD
Lopez Cazaux 2019 Eur Arch Paediatr Dent [25]	Nr. of participants: 52 Mean age: 10.2 y.o. Target population: ASD	Aim: evaluate effectiveness of toothbrushing training program using iPad app through visual pedagogy and behavioral approach	Intervention: “çATED” app Dental care activity: toothbrushing App characteristics: pictograms/photos to divide brushing steps Duration: 240 days	Engagement: passive Usage frequency: MD Average session duration: MD Usability ratings: MD	Main result: the use of the app improved toothbrushing. Child level: more autonomy was achieved, and fewer toothbrushing steps were omitted. Caregiver level: MD. Dentist level: no children performed all the steps alone without assistance. Total failures in brushing decreased progressively during follow-ups.
Narzisi 2020 Brain Sci. [28]	Nr. of participants: 59 Mean age: 9.9 y.o. (range 4–16 y.o.) Target population: ASD	Aim: improve access to dental care, familiarization with dental setting, and cooperation	Intervention: web app “My Dentist” combined with ICT Dental care activity: oral health education, professional oral hygiene, sealing, restorative treatment App characteristics: video, social stories and personalized games and activities Duration: 180 days	Engagement: Interactive Usage frequency: MD Average session duration: MD Usability ratings reported: MD	Main result: The combination of the web app with ICT improved access to dental care, cooperation during treatments, and oral hygiene status. Child level: an improvement in collaboration and behavior. Caregiver level: the app was well received and accepted. Dentist level: the personalization of the app and ICT contents improved collaboration during treatments.
Tan 2024 Autism. [26]	Nr. of participants: 15 Mean age: age range 3–12 y.o. Target population: ASD children	Aim: improve resources to support caregivers during oral hygiene of ASD children	Intervention: app Dental care activities: oral health education, familiarization with dental setting App characteristics: social stories, educational content, positive reinforcement with rewards, personalized messages, and calendar Duration: 21 days	Engagement: passive Usage frequency: MD Average session duration: MD Usability ratings: easy to use and esthetically pleasing (self-reported)	Main result: an improvement in the awareness of the importance of oral health and care. Child level: MD. Caregiver-level: the app was well received and useful. Dentist-level: MD.

Abbreviations: “nr.”—number; “y.o.”—years old; “MD”—missing data; “ASD”—autism spectrum disorder; and “ICT”—information and communication technologies.

**Table 2:** Characteristics of mobile applications tailored for children with Down syndrome: population, methods, intervention, engagement and usability, outcomes, feedback, and sustainability.

Study	Population	Methods	Intervention	Engagement and Usability	Outcome(s), Feedback, and Sustainability
Pamungkas 2019 J Int Dent Med Res [27]	Nr. of participants: 34 Mean age: age range 7–12 y.o. Target population: Down syndrome	Aim: improve the understanding of oral health education using a mobile app	Intervention: “Gigiku Sehat” app Dental care activities: oral health education App characteristics: animated videos and interactive games for oral hygiene practices, educational games and quizzes to test knowledge, digital rewards Duration: 7 days	Engagement: interactive Usage frequency: once a day Average session duration: 15 min Usability ratings: easy to use (self-reported)	Main results: the app was effective in improving oral health knowledge in children with Down syndrome Child level: the app improved oral health knowledge Caregiver level: the app was easy to use and engaging Dentist level: MD

Abbreviations: “Nr.”—number; “y.o.”—years old; and “MD”—missing data.

## Data Availability

Reported data are available on the MEDLINE/PubMed, Scopus, and Web of Science databases.

## Abbreviations

The following abbreviations are used in this manuscript:

m-health	Mobile health
SHCN	Special healthcare needs
ADHD	Anxiety, attention deficit, and hyperactivity disorder
ASD	Autism spectrum disorder
WHO	World Health Organization
ICT	Information and communication technologies
